# Controlling chaos by the system size

**DOI:** 10.1038/s41598-021-87233-8

**Published:** 2021-04-22

**Authors:** Mahdi Ghadiri, Rouslan Krechetnikov

**Affiliations:** Department of Mathematics, University of Alberta, AB T6G 2R3 Edmonton, Canada

**Keywords:** Nonlinear phenomena, Fluid dynamics, Applied mathematics

## Abstract

Despite the ubiquity of physical systems evolving on time-dependent spatial domains, understanding their regular and chaotic dynamics is still in a rudimentary state. While chaos implies that the system’s behavior can be altered by small perturbations, this sensitivity proves to be useful for control purposes. Here we report on the experimental discovery of a novel mechanism to control chaos by time-variation of the system (spatial domain) size: depending upon the rate of the latter, the chaotic state may be completely prevented. Our experimental observations are disentangled with theoretical insights and numerical modeling, which also reveals the ability to control spatio-temporal chaos, thus making the findings relevant to a wide range of natural phenomena.

## Introduction: Faraday wave phenomena

**Figure 1:**
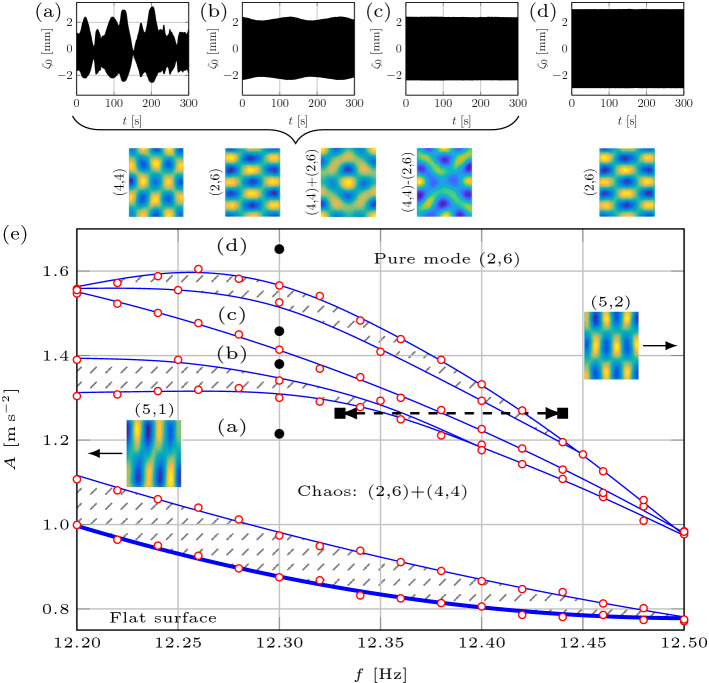
A roadmap. Interactions between modes (2, 6) and (4, 4) on the time-fixed domain of dimensions $L \times W = 120 \, {\mathrm {mm}} \times 150 \, {\mathrm {mm}}$. Surface deformations $\zeta _{0}(t)$ (**a**–**d**) corresponding to solid circles in the amplitude-frequency (*A*, *f*) map (**e**). Four distinct regions exist above the threshold (thick curve): the chaotic (**a**), the periodic time-dependent (**b**) and -independent (**c**) pattern competitions, and time-independent periodic pure mode (2, 6) in (**d**). Below the panels are the patterns observed when the respective $\zeta _{0}(t)$ was recorded: (2, 6) in (**e**) and all of (4, 4), (2, 6), $(2,6)+(4,4)$, $(2,6)-(4,4)$ in (**a**–**c**). The measurement error of the amplitude *A* is $10^{-2} \, {\mathrm {m \, s^{-2}}}$.

There exist numerous physical systems evolving on time-dependent spatial domains^1^—ranging from crystal growth, pattern formation on animal skin, Hydra’s tentacles, whorled leaves, teeth primordia in the alligator to quantum particles traveling in a time-evolving potential and galaxy agglomeration in the expanding Universe—many of which exhibit chaotic dynamics. To illustrate how one can traverse the edge of chaos by varying the domain size, as a testbed we have chosen the Faraday waves phenomenon^2^, which is a paradigmatic example in pattern-forming systems, known to exhibit temporal chaos as well. When a container is filled with a liquid and vibrated with sufficient acceleration *A* in the direction of gravity, standing surface waves, historically named after Faraday^2^, are formed and oscillate at a frequency $\omega _{0}/2\pi$, half that of the forcing *f*. Such waves exhibit patterns with a large variety of shapes and symmetries (Fig. 1), depending on the fluid properties, layer depth, driving and boundary conditions. Our experimental setup (cf. “Methods”) produces Faraday waves and enables time-dependent variation of the rectangular container length $L(t) = L_{0} + v \, t$ at a wall speed (rate) *v*/2 with the help of computer controlled stepper motors while still maintaining the container width *W* and liquid layer depth *h* constant.

In the rectangular domain $(x,y) \in [-L/2,L/2] \times [-W/2,W/2]$, the surface deformation due to excitation of the single mode $l=(m,n)$ is of the form

1 $$\begin{aligned} \zeta (t,x,y)=a_{l}(t,\tau ) \, S_{l}(x,y), \end{aligned}$$

where $S_{l}(x,y)=\cos{\pi m \left(x/L +1/2\right)} \cos{\pi n \left(y/W + 1/2\right)}$ is the spatial pattern of amplitude $a_{l}(t,\tau)=C_{l}(\tau) \cos{\left[\omega_{0} t + \phi_{l}(\tau)\right]}$; $C_{l}(\tau )$ and $\phi _{l}(\tau )$ are the wave amplitude and phase evolving on a slow timescale $\tau \gg 2\pi /\omega _{0}$; *m* and *n* represent the number of half-wavelengths formed in each direction^3,4^.

Simultaneous excitation of two modes at the same values of driving amplitude-frequency (*A*, *f*) takes place in the overlap region of the two stability curves commonly known as the pattern competition regime^5^. In this regime slow amplitudes $C_{l}(\tau )$ of two different patterns $l_{1}$ and $l_{2}$ are both nonzero, non-equal and oscillate with different phases at a frequency smaller than the driving one by more than two orders in magnitude^6,7^. According to (1), at the centre of the container with the addition of meniscus waves of the amplitude $a_{M}(t,\tau )= C_{M}(\tau ) \cos {\left[ 2\omega _{0} t+\phi _{M}(\tau )\right] }$, we have

2 $$\begin{aligned} \zeta _{0}(t) \equiv \zeta (t,0,0) = a(t,\tau )+a_{M}(t,\tau ), \end{aligned}$$

where $a(t,\tau )= a_{l_{1}}(t,\tau ) + a_{l_{2}}(t,\tau ) \equiv C(\tau ) \cos {\left[ \omega _{0} t+\phi (\tau )\right] }$ is the superposition of the two Faraday modes $l_{1}=(2,6)$ and $l_{2}=(4,4)$. From measured $a(t,\tau )$ the amplitude envelope $C(\tau )$, wave frequency $\omega _{0}$, and phase $\phi$ are recovered and, accordingly, the Faraday waves complex slow amplitude $U(\tau ) = C(\tau ) \, {\mathrm {e}}^{- i \phi (\tau )}$ is reconstructed. Using its real part ${\text {Re}}U$ as the time series, the state of the dynamical system, either periodic or chaotic, is determined by calculating the fractal dimension ${\mathscr {D}}$ of the chaotic attractor, with a non-integer ${\mathscr {D}}$ corresponding to a chaotic state, whereas ${\mathscr {D}}=1$ to a periodic one (cf. “Methods”).

To navigate the experiments on time-dependent domains, first we developed the roadmap on the time-fixed domain analogous to that in the literature^8^ in the (*A*, *f*)-space surrounding the pattern competition regime of the modes $l_{1}$ and $l_{2}$, cf. Fig. 1e. This map is limited from the left and right by the excitation of nearby modes (5, 1) and (5, 2), respectively, and defines the boundaries between chaotic and periodic regimes, which we will attempt to traverse using domain deformation *L*(*t*). Four distinct regions are observed above the threshold (thick curve): (a) chaotic competition between the two modes; (b) periodic competition with a time-dependent envelope; (c) periodic competition with a time-independent envelope; and (d) the pure mode (2, 6) oscillating periodically with a time-independent envelope. The shaded areas represent the hysteretic regions: if one starts from below the threshold curve and increases the amplitude, in the lowest shaded area a flat surface is observed, but if the starting point is in region (a), then by decreasing the amplitude and entering the shaded area, chaotic competition is exhibited. The surface patterns presented in Fig. 1a-d are observed during the record of the corresponding surface deformation $\zeta _{0}$ at various instances in time, i.e. mode (2, 6) in region (d) and all of the combinations (4, 4), (2, 6), $(2,6)+(4,4)$, $(2,6)-(4,4)$ in (a–c), because in any of the latter regions the two modes are competing.

**Figure 2:**
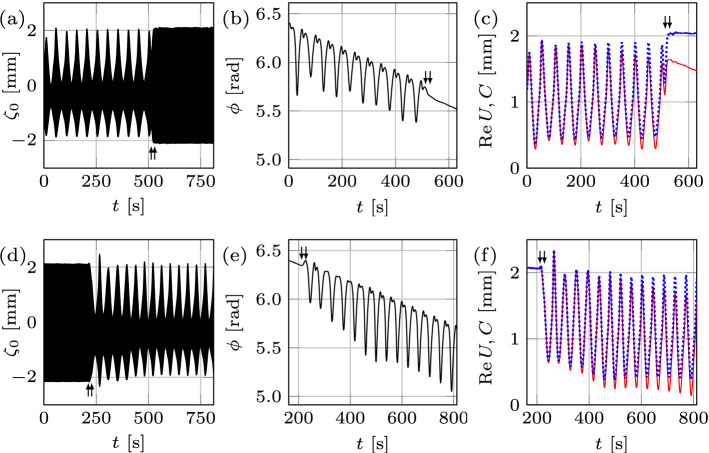
Regime change in response to domain deformation at $f=12.33 \, {\mathrm {Hz}}$ and $A=1.26 \, {\mathrm {m \, s^{-2}}}$. Top (bottom) row represents domain growth (shrinkage) by $\Delta L = 2\, {\mathrm {mm}}$ at a rate $0.06 \, {\mathrm {mm \, s^{-1}}}$, over which the regime changes from chaotic (periodic) to periodic (chaotic). (**a**, **d**) The surface deformation $\zeta _{0}(t)$ within the arrows indicates the start and finish of wall motion. (**b**, **e**) The slow phase $\phi$. In (**c**, **f**) the amplitude envelope *C* and the real part of the slow amplitude ${\text {Re}}U$ are shown by blue dotted and red solid curves, respectively.

## Results: experiments

### Traversing the edge of chaos by changing the system size

**Figure 3:**
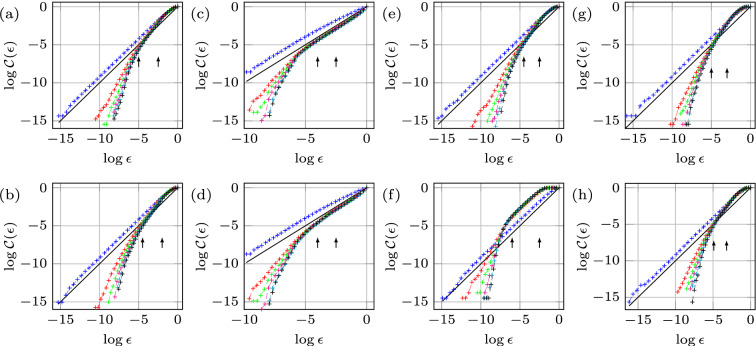
The correlation function ${\mathcal{C}}(\epsilon )$ obtained for each physical experiment from a single time series of the real part of the slow amplitude ${\text {Re}}U$ for various embedding dimensions *m*. The solid black line with slope one is shown to compare with the saturated slope ${\mathscr {D}}$ in the scaling region (between the two arrows). Panels (**a**, **b**), (**e**, **f**), and (**g**, **h**) indicate the chaotic strange attractor dimension ${\mathscr {D}}=1.38$, 1.42, 1.52, 1.00, 1.48, and $1.31\pm 0.02$, corresponding to Figs. 2a,d, 4a,d, and 5a,d, respectively. In (**c**) the correlation function for 100 fast cycles prior to wall motion in Fig. 2d is presented indicating regular periodic dynamics with ${\mathscr {D}}=1.00\pm 0.02$, whereas in (**d**) inclusion of 65 additional fast cycles corresponding to the interval of domain shrinkage $\delta L = 1.3 \, {\mathrm {mm}}$ resulted in the fractal dimension ${\mathscr {D}}=1.1\pm 0.02$ indicating the transition into chaos.

We start by illustrating how domain growth can lead to regularization of the chaotic regime corresponding to $f=12.33 \, {\mathrm {Hz}}$, $A=1.26 \, {\mathrm {m \, s^{-2}}}$, and $L= 120 \, {\mathrm {mm}}$, cf. Fig. 2a–c. Despite that ${\text {Re}}U$ may appear periodic (Fig. 2c), its analysis reveals a chaotic attractor with fractal dimension of ${\mathscr {D}}=1.38$ (Fig. 3a). At $t=513 \, {\mathrm {s}}$ the domain starts to grow at a rate $0.06 \, {\mathrm {mm \, s^{-1}}}$ and in the course of stretching for $2\, {\mathrm {mm}}$ the system reaches the periodic state, where mode (2, 6) oscillates periodically with a time-independent envelope (Fig. 2a,c). Several initial *key observations* can be made from Fig. 2a–c. *First*, the fractal dimension ${\mathscr {D}}$ decreases with increasing domain size as opposed to, say, Rayleigh–Bénard convection^9,10^ in which ${\mathscr {D}}$ increases with the system size; in general, however, the trend can be reversed depending upon the interplay between the bulk and boundary dynamics. *Second*, using the initial phase and envelope within the time-independent periodic pure mode regime as the reference, during the domain growth (Fig. 2b,c) the regime transformation is accompanied by small changes in the slow phase ($-0.9\%$) and noticeable variations in the envelope ($36.1\%$), thus indicating the amplitude chaos with no phase-slips. *Third*, in this case the transition from chaotic to periodic state takes place during the wall motion (indicated by arrows) over $\delta L = 1.68 \, {\mathrm {mm}}$, which is smaller than the total domain size variation $\Delta L = 2\, {\mathrm {mm}}$; that is, the amplitude $a(t,\tau )$ reaches constant envelope over $\delta L$, and once the wall motion ceases at $\Delta L$ the system is already oscillating periodically with a time-independent envelope. However, this scenario is not universal: in other experiments, e.g. in Fig. 11a with an initial chaotic state of dimension ${\mathscr {D}}=1.82$ undergoing domain growth at a rate $0.15 \, {\mathrm {mm \, s^{-1}}}$, the transition to periodic regime is not confined to the domain deformation time interval. This difference is likely due to a weaker chaotic state in Fig. 2a–c (${\mathscr {D}}=1.38$) compared to that in Fig. 11a (${\mathscr {D}}=1.82$), which is characterized by more unstable (in terms of Lyapunov exponents) periodic orbits^11^ and therefore requires a longer transition regime in order to regularize the system. Indeed, on the one hand, since a chaotic set, on which the trajectory of the chaotic process lives, has embedded within it a large number of unstable low-period orbits, sensitive dependence on small changes to the chaotic state implies that the system’s behavior can be altered by using smaller (domain) perturbations for larger ${\mathscr {D}}$. On the other hand, due to higher sensitivity at larger ${\mathscr {D}}$, probabilistically it takes longer to ‘hit’ a periodic orbit possessing a sufficiently large basin of attraction.

Next, using the periodic state reached at $L= 122 \, {\mathrm {mm}}$ as the initial state and then shrinking the domain back to the original length $L= 120 \, {\mathrm {mm}}$ (Fig. 2d–f) with the same wall speed returns the system to the chaotic state, but with a fractal dimension ${\mathscr {D}}=1.42$ (Fig. 3b). The reconstructed slow phase $\phi$ (Fig. 2e) and envelope *C* (Fig. 2f) indicate that qualitatively the dynamical system is experiencing a process reverse to that during the domain growth. The transition $\delta L$ from periodic to chaotic state is also shorter in length than the domain shrinkage interval $\Delta L$: analysis of the amplitude $a(t,\tau )$ reveals that before the start of domain shrinkage, the system is in a periodic state with ${\mathscr {D}}=1.00 \pm 0.02$ (Fig. 3c), while the domain shrinkage $\delta L= 1.3 \, {\mathrm {mm}}$ leads to ${\mathscr {D}}=1.1$ (Fig. 3d), hence indicating a transition to chaos before the walls come to rest. In comparison with the domain growth cases presented in Fig. 2a ($\delta L= 1.68 \, {\mathrm {mm}}$) and Fig. 11a ($\delta L= 2 \, {\mathrm {mm}}$), domain shrinkage (Fig. 2d) leads to regime transformation on a smaller domain size variation $\delta L$, which is the *fourth* key observation to be interpreted below, in the context of numerical study.

### The effect of the domain evolution rate: chaos prevention

With the help of the same map (Fig. 1e), another remarkable ability of domain evolution to control chaos is identified: namely, isolating one of the competing modes in the regime, which on a time-fixed domain of the same size would otherwise correspond to a chaotic pattern competition. As evident from Fig. 1e, the system at $f=12.33 \, {\mathrm {Hz}}$ and $A=1.26 \, {\mathrm {m \, s^{-2}}}$ is located in the chaotic competition regime—we examined if this final state of the system could be altered with the help of domain evolution at varying rates. These experiments are similar to the one in Fig. 2d–f but conducted at different evolution rates. Starting with the pure mode (2, 6) oscillating periodically with a time-independent envelope on the domain $L= 122\, {\mathrm {mm}}$ we can see if shrinking the domain to $L= 120\, {\mathrm {mm}}$ at different rates would provide the ability to control the final state of the system, which is known to be chaotic on a time-fixed domain of the same size.

**Figure 4:**
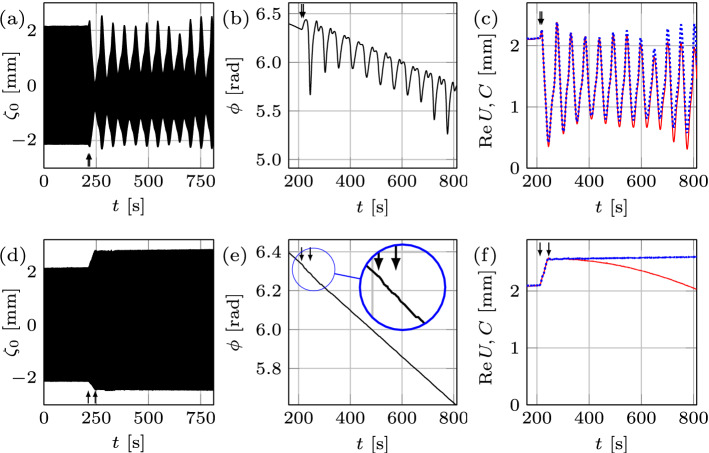
Wall speed effect at $f = 12.33 \, {\mathrm {Hz}}$ and $A = 1.26 \, {\mathrm {m \, s^{-2}}}$. The slower evolution of the domain prevents the system from entering the chaotic regime. The domain shrinkage $\Delta L=2\, {\mathrm {mm}}$ starts at $L= 122\, {\mathrm {mm}}$ with two wall speeds 0.15 (top) and $0.03 \, {\mathrm {mm \, s^{-1}}}$ (bottom).

Top and bottom rows in Fig. 4 show two different runs at wall speeds of 0.15 and $0.03 \, {\mathrm {mm \, s^{-1}}}$, respectively. At $0.15 \, {\mathrm {mm \, s^{-1}}}$, the domain shrinkage leads to the chaotic pattern competition (Fig. 4a)—the regime expected on a time-fixed domain of the same size—with the strange attractor of dimension ${\mathscr {D}}=1.52$ (Fig. 3e). Surprisingly, decreasing the speed below $0.03 \, {\mathrm {mm \, s^{-1}}}$ prevents the system from entering the chaotic regime (Fig. 4d), and makes it continue with periodic oscillation of mode (2, 6) even after $535 \, {\mathrm {s}}$ from the moment the walls are brought to rest. That is, domain deformation isolates mode (2, 6) out of the expected chaotic pattern competition regime on a time-fixed domain of the same size; this fact is confirmed by the corresponding attractor dimension ${\mathscr {D}}=1.00\pm 0.02$ in Fig. 3f. The two other experiments (not presented here) performed at the speeds 0.3 and $0.015 \, {\mathrm {mm \, s^{-1}}}$ reinforced the above result, i.e. the higher speed led to the chaotic state while the lower one allowed the system to remain periodic. Together with another domain shrinkage experiment at the wall speed $0.06 \, {\mathrm {mm \, s^{-1}}}$ presented in Fig. 2d–f one can conclude that the threshold wall speed lies in the transition interval $0.03-0.06 \, {\mathrm {mm \, s^{-1}}}$. Therefore, if slow enough, the domain deformation prevents the system from entering the chaotic regime—the signature of hysteretic behavior, which can be understood as follows.

Water surface profiles reveal that domain shrinkage at slow rates only shortens the wavelength $\lambda _{x}$ of mode (2, 6), which is compensated by the increase in wave amplitude (Fig. 4d) in accordance with the mass conservation $C \times \lambda _{x} \times \lambda _{y} \approx {\mathrm {const}}$. Thus, slow domain evolution is not strong enough to perturb the system away from the periodic state of mode (2, 6), i.e. the system is capable of adapting and thus staying near this periodic orbit; therefore, mode (4, 4) is no longer formed. On the contrary, during a rapid domain evolution the system experiences an instability leading to the appearance of mode (4, 4) and hence competition with the preexisting mode (2, 6) resulting in the chaotic regime. At the transitional wall speeds, the system traverses the edge of chaos, which separates the basins of attraction where perturbations evolve either towards the regular or chaotic regime.

From the dynamical systems perspective, domain evolution is able to force the trajectory of the dynamical system under consideration closer to one of its fixed points thus reaching or remaining in a stable periodic state. From the physical point of view, on the other hand, this effect is related to the bulk flow structure, which not only could be different for identical Faraday wave patterns^12^, but also may be altered by wall motion. Namely, the well-developed flow on a fixed domain might differ from the flow formed when walls move to the same domain size, leading to the hysteresis, i.e. the dependence of the final Faraday wave pattern on the dynamical system trajectory.

### Relation to frequency chirping

**Figure 5:**
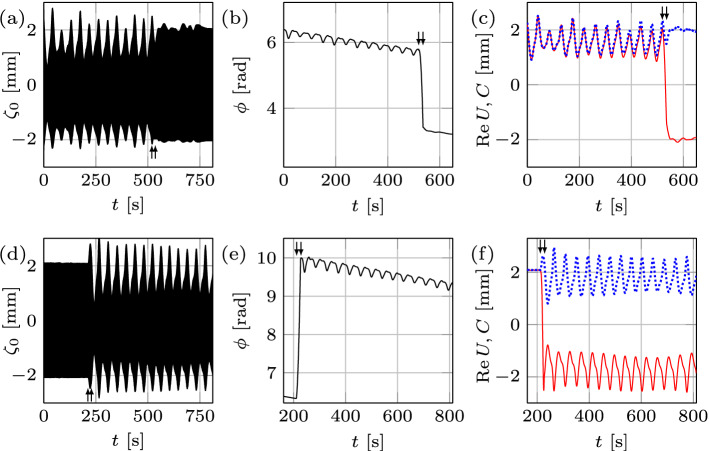
Regime change in response to frequency chirping. Frequency change $12.33 \, {\mathrm {Hz}} \Leftrightarrow 12.44 \, {\mathrm {Hz}}$ takes place on a fixed domain of size $L= 120\, {\mathrm {mm}}$. The top (bottom) row represents frequency increase (decrease) for $0.11\, {\mathrm {Hz}}$ at the rate of $0.0066 \, {\mathrm {Hz \, s^{-1}}}$, due to which the regime changes from chaotic (periodic) to periodic (chaotic).

Our study would be incomplete without experimental comparison of domain deformation effects to that due to frequency variation (chirping) in time, since these two methods to control dynamics may seem to be interrelated. Indeed, from the dispersion relation $\omega _{0}^{2} = [g k + (\sigma /\rho ) k^{3}] \tanh {k h}$ for linear Faraday waves, one can see that the variation of the frequency $\omega _{0}(t)$ in time affects the instability wavenumber $k(t)=|{\mathbf {k}}|$; hence, if the number of pattern cells is not changing under the domain deformation, but the wavelength is being adjusted instead, domain shrinkage leading to wavenumber increase $k\uparrow$ should be equivalent to frequency increase $\omega _{0}\uparrow$ and vice versa. However, due to the Eckhaus instability of the modes and because of the mode quantization on a finite size domain^1^, the link between domain deformation and frequency chirping is not as monotonic as one may glean from the roadmap in Fig. 1. The phenomena similar to that in Fig. 2 can be investigated with frequency being the controlling parameter, while the domain size is fixed at $L= 120\, {\mathrm {mm}}$ (Fig. 5). To be able to compare the two processes, the initial starting point on the roadmap (Fig. 1) should be the same. In order to reveal the effects of frequency variation, it is desired to pass through all regions (a–d) in Fig. 1, from a chaotic to a periodic state of the pure mode (2, 6) and vice versa. This is achieved, cf. Fig. 1e, at $A=1.26 \, {\mathrm {m \, s^{-2}}}$ and *f* varying from 12.33 to $12.44 \, {\mathrm {Hz}}$ at the rate of $0.0066 \, {\mathrm {Hz \, s^{-1}}}$, thereby taking the same amount of time as the domain deformation (Fig. 2).

Considering that in Fig. 5a the location of the arrows indicates the start and finish of the frequency chirping, it is evident that, unlike the domain deformation case in Fig. 2, the transition regime is extended far beyond the point where the frequency increase ceases: the envelope takes a considerably longer time (about $250\, {\mathrm {s}}$) to become time-independent and thus the regular regime to settle compared to the frequency chirping application time. Since the chaotic attractor dimensions in these two cases are comparable (${\mathscr {D}} = 1.38$ inf Fig. 3a and ${\mathscr {D}} = 1.48$ in Fig. 3g, respectively), this likely means that the system adapts better to domain size variation than to frequency chirping. To that end, Fig. 5b,c demonstrate relative changes in slow phase $\phi$ and the envelope *C* of $-36.6\%$ and $40.7\%$ in the course of frequency chirping, respectively. It seems that this substantial change in the phase is the reason behind the extended transition in the frequency chirping case, whereas the corresponding phase change in the domain deformation case in Fig. 2b is negligible and correlated to a short transition stage contained within the wall motion interval. It must be noted that the observed phase jumps $-0.74 \pi$ in Fig. 5b and $1.1 \pi$ in Fig. 5e are not phase-slips (requiring $\Delta \phi = 2 \pi$) since they result from the appearance of a new mode (4, 4) in addition to pre-existing one (2, 6) thus leading to a change in the total phase of both superimposed modes (2). This behavior differs from the standard frequency chirping when the driving frequency *f* crosses the border of the Arnold tongue leading to the loss of synchronization (zero detuning) between the driving and oscillator frequencies and thus to infinite growth of the phase difference^13^. This growth is not uniform: there are epochs when the phase difference is nearly constant, and other, much shorter, epochs where the phase difference changes relatively rapidly by $2 \pi$, corresponding to a phase-slip. On the other hand, phase jumps, which are not multiples of $2 \pi$, are known to accompany phase synchronization transitions in chaotic systems which exhibit phase coherence^14^ as in our case, cf. Figs. 2b,e, 5b–e, and 4b. While the phase coherence of a chaotic attractor may mean that a suitably defined phase increases steadily in time, here we understand the phase coherence in a broader sense when the phase exhibits some pattern on a long timescale—the fact that chaotic systems, if examined over a sufficiently long time, can display regular patterns is known^15^ and could be the result of some symmetries underlying the dynamical system^16^.

The lower panels in Fig. 5 correspond to the reverse process, i.e. decrease of the frequency leading to the change of the regime from periodic to chaotic (${\mathscr {D}} = 1.31$ in Fig. 3h). Since the system reaches the chaotic state once the frequency decrease is finished (Fig. 5d), the transition regime must be contained within the frequency chirping interval. Compared to domain shrinkage (Fig. 2f), the envelope *C* in Fig. 5f (dotted curve) experiences a sharp increase during the very initial stage of frequency decrease, i.e. at the location of the first arrow. This is because in addition to mode (2, 6) a new appearing mode (4, 4) superimposes thus increasing the total amplitude *C* of both modes. In general, while frequency chirping leads to a “domain flow” linear term in the corresponding amplitude equation^17^ similar to that for the actual domain flow^18^ in equation (3) since both of them amount to the Doppler-like effect, at the nonlinear level there are crucial differences in the corresponding amplitude equations and thus in the finite-amplitude behaviors between frequency chirping and domain deformation reported above.

Finally, in the context of the frequency chirping discussion, it should be mentioned that changing the waveform of excitation provides yet another degree of control of the transition between regular and chaotic regimes as demonstrated, for example, in the studies of bouncing states of a droplet on a liquid surface^19^. While it is known that changing the forcing waveform from sinusoidal to square and triangular does not qualitatively affect the resonant tongue structure^20^, its effect on the transition from regular to chaotic regimes and an interplay with varying the system size require a separate study.

## Results: theory

### The Ginzburg–Landau model

Spatially extended (and thus infinite-dimensional) systems are prone not only to temporal (amplitude) chaos, which is well studied in finite-dimensional contexts, but also to spatio-temporal chaos^21,22^, in particular phase turbulence^23,24^. Amplitude chaos is often characterized by the occurrence of phase slips during which the wave amplitude goes to zero (defect) and the total phase changes by $2 \pi$, i.e. a wavelength is inserted or eliminated; in the phase-chaotic regime essentially no phase slips occur^25^.

To gain further insights into controlling chaos by variation of the domain size, as a minimal model we study the Ginzburg-Landau equation (GLE) universally valid near critical points of pattern forming systems^26^. Although the Faraday wave phenomenon is two-dimensional and would require two coupled GLEs to describe the two cross-roll hydrodynamic system^27^, its qualitative understanding can still be achieved using a single 1D complex GLE (cGLE) evolving on the slow time $\tau$ and long spatial $x \in [-L(\tau )/2,L(\tau )/2]$ scales written here in a non-dimensional form^22^:

3 $$\begin{aligned} U_{\tau } = (1 - {\mathrm {i}} {\fancyscript {u}})\,U + (1 + {\mathrm {i}} \alpha ) \,U_{xx} -(1 + {\mathrm {i}} \beta )\, |U|^{2} \, U, \end{aligned}$$

which incorporates an extra term ${\mathrm {i}} {\fancyscript {u}} U$ accounting for the effects of advection and dilution^18^ due to domain evolution $L(\tau ) = L_{0} + v \, \tau$ at a (non-dimensional) rate *v*; here ${\fancyscript {u}}(\tau ,x)$ represents the domain velocity at point *x*. Chaotic behavior exhibited by the cGLE on time-fixed domains has been extensively studied^28^ and will be used as a reference. Depending on the values of $\alpha$ and $\beta$, the cGLE on time-fixed domains can exhibit plane waves, spatio-temporal chaos, and intermittency^22,28^. The chaotic regime occurs beyond the Benjamin-Feir-Newell curve $\beta \le -\alpha ^{-1}$, leading either to phase chaos, defect chaos, or bichaos^22,28^. When *U* has no zeros, |*U*| remains saturated and only its phase will be dynamically active, leading to phase chaos. On the contrary, if *U* vanishes at some point *x*, then the complex phase is undefined there and a phase slip occurs leading to defect chaos. In the bichaotic regime, taking place for the values of $\alpha$ and $\beta$ closer to the Benjamin–Feir–Newell curve $\alpha \beta =-1$, defect- and phase-chaotic attractors coexist^22^.

**Figure 6:**
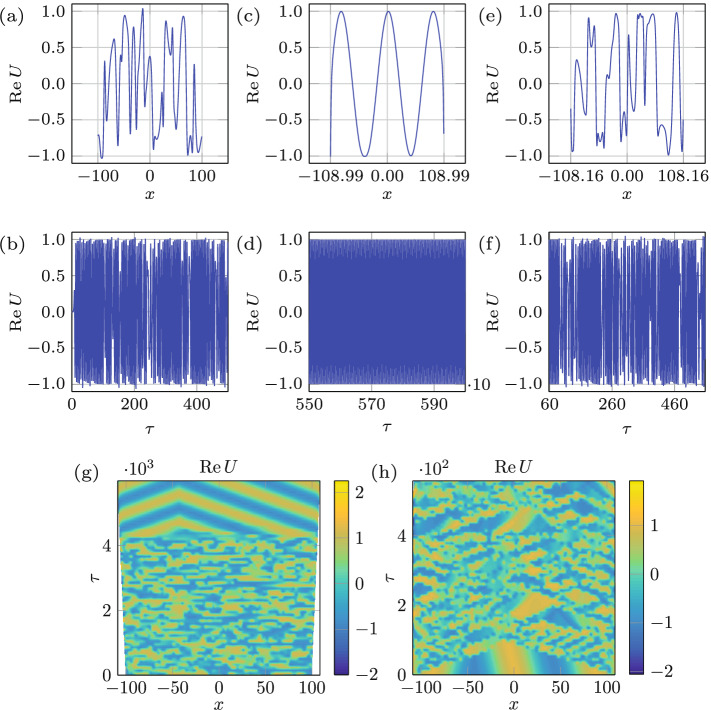
Regime change in response to domain deformation. (**a**, **b**) The spatio-temporal bichaos used as the initial state with domain size fixed at $L= 200$ and $(\alpha ,\beta )=(0.75,-1.4)$. In (**c**, **d**) the state of the system after domain growth by $\Delta L=17.98$ at a rate $v=0.003$, over which the regime changes from chaotic to periodic. Starting from the periodic state in (**c**, **d**), shrinking the domain by $\Delta L=1.66$ at a rate $v=0.003$ leads to the chaotic state (**e**, **f**). In (**a**, **c**, **e**) ${\text {Re}}U(x,\tau )$ is plotted at the corresponding final integrated times $\tau _{f} = 500, \, 6000$ and 558, respectively, whereas in (**b**, **d**, **f**) ${\text {Re}}U(x,\tau )$ is plotted at $x=0$ over $\tau _{f}-500\le \tau \le \tau _{f}$. Space-time plots of the growth (**g**) and shrinkage (**h**) indicate the transition happens over $\delta L=5.40$ and $\delta L=0.29$, respectively.

### Traversing the edge of chaos

**Figure 7:**
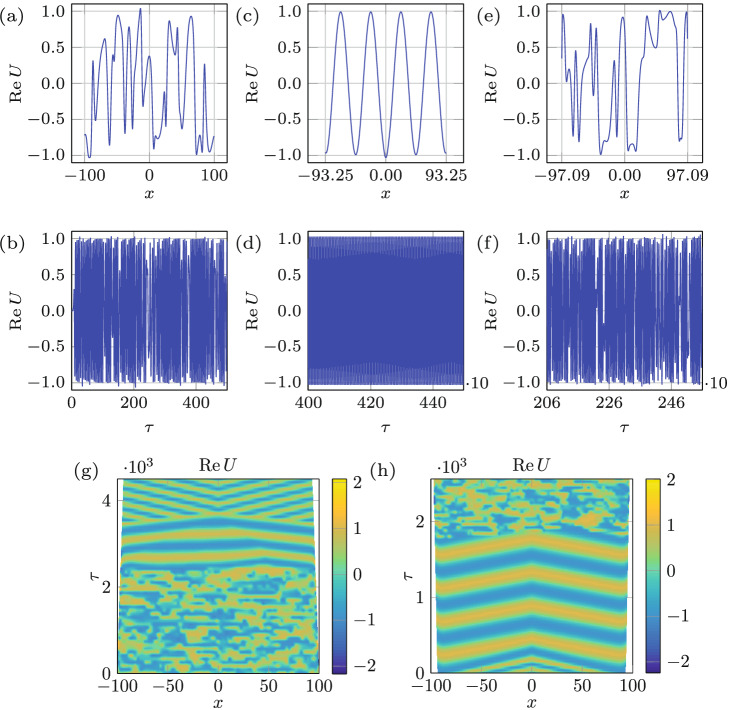
Regime change in response to domain deformation. (**a**, **b**) depict the spatio-temporal bichaos used as an initial state with domain size $L= 200$ and $(\alpha ,\beta )=(0.75,-1.4)$. In (**c**, **d**) the state of the system after domain shrinkage by $\Delta L=13.50$ at a rate $v=0.003$, due to which the regime changes from chaotic to periodic, is presented. Starting from the periodic state in (**c**, **d**), growing the domain by $\Delta L=7.68$ at a rate $v=0.003$ leads to the chaotic state (**e**, **f**). Space-time plots of the shrinkage (**g**) and growth (**h**) processes indicate the transition happens over $\delta L=5.04$ and $\delta L=5.64$, respectively.

We first consider domain shrinkage and its reverse process in analogy with experiments in Fig. 2. Figure 6a,b depict the spatio-temporal bichaotic state on a time-fixed domain—resulting from integration of (3) with $(\alpha ,\beta )=(0.75,-1.4)$ up to $\tau _{f}=500$—used as the initial condition for integration on a time-varying domain. Then the domain growth by $\delta L=17.98$ at the rate of $v=0.003$ brings the system to the periodic state, for which the transition itself (measured here between the last moment a given regime is observed across the entire spatial domain to a similar moment when a new regime is settled across the entire domain as well) takes place only over ${\delta L}=5.40$ (Fig. 6g). Figure 6c,d present ${\text {Re}}U(x,\tau _{f}=6000)$ and ${\text {Re}}U(x=0,\tau )$ where $\tau _{f}-500\le \tau \le \tau _{f}$, respectively, and indicate that the system has reached periodic state both in time and space, thereby establishing the capability of domain evolution not only to control the temporal but also the spatial chaos. To examine the reversibility of the process, the domain growth is undertaken starting from the periodic state in Fig. 6c,d—as demonstrated in Fig. 6h, the system becomes chaotic right away, though not across the entire domain, and remains chaotic even beyond $\tau =558$ for which the final stage is presented in Fig. 6e,f. Therefore, the regime transformation from chaotic to periodic is reversible, though the required change of the domain size during shrinkage and growth are not necessarily equal or even of the same order (Fig. 6g,h). To confirm that domain shrinkage also has the capability to control chaotic state, numerical investigation a similar to the above was performed in Fig. 7, except that the domain now first undergos shrinkage and then growth. Part (g) of that figure indicates that the transition from the chaotic to periodic regime takes place over the domain shrinkage by $\delta L=5.04$, while the domain growth by $\delta L=5.64$ takes the system back to the chaotic state (h).

An important observation from the results of integration in Figs. 6 and 7, both initiated from an identical state (panels a and b in these figures), is that compared to domain growth, shrinkage transforms the regime over a smaller change in the domain size, i.e. $\delta L=0.29$ in Fig. 6h and $\delta L=5.04$ in Fig. 7g vs. $\delta L=5.40$ in Fig. 6g and $\delta L=5.64$ in Fig. 7h. To explain this asymmetry between growth and shrinkage, which was also observed experimentally in Fig. 2, we recall that compared to domain growth shrinkage causes early phase-slips, as known theoretically^1^ in 1D and experimentally in 2D^29^, thereby leading to a faster change in the wavenumber structure of a pattern. Hence, if the route to chaos requires the change in the wavenumber structure of the original periodic mode, then the difference in the phase-slip occurrence characteristic times between domain shrinkage and growth is responsible for the observed asymmetry. If, on the other hand, the route to chaos from a given original periodic mode simply requires an excitation of another mode, the interaction of which with the original one leads to temporal chaos (so-called pattern competition)^6^, then the difference lies in the subcritical nature of the transition to chaos, which can occur directly. The latter happens in other contexts, e.g. when a modulationally destabilized monochromatic wave in a fluid system undergoes a subcritical bifurcation directly into chaos, provided dissipation is weak enough^30^. It is known that finite amplitude perturbations are needed in order to trigger subcritical instabilities^31,32^. In the context of Faraday waves, such finite-amplitude perturbations are always present, e.g. in our system due to meniscus waves and wall motion when domain size is varied. The direct transition from periodic to chaotic via subcritical bifurcation is justified not only by the periodic and chaotic regions being adjacent^33^ in the amplitude-frequency (*A*, *f*)-plane, but also by the fact that superposition of modes, where chaos is observed, belongs to the subcritical side of the resonant tongue^8^. Given that in the case of domain shrinkage modes from the subcritical side of the resonant tongue are excited via a finite-amplitude instability, while in the case of growth modes from the supercritical side emerge, one indeed expects irreversibility and therefore hysteretic behavior of Faraday waves^29^, which is fundamentally due to viscous dynamics of the bulk flow underlying the surface pattern^12,34^.

**Figure 8:**
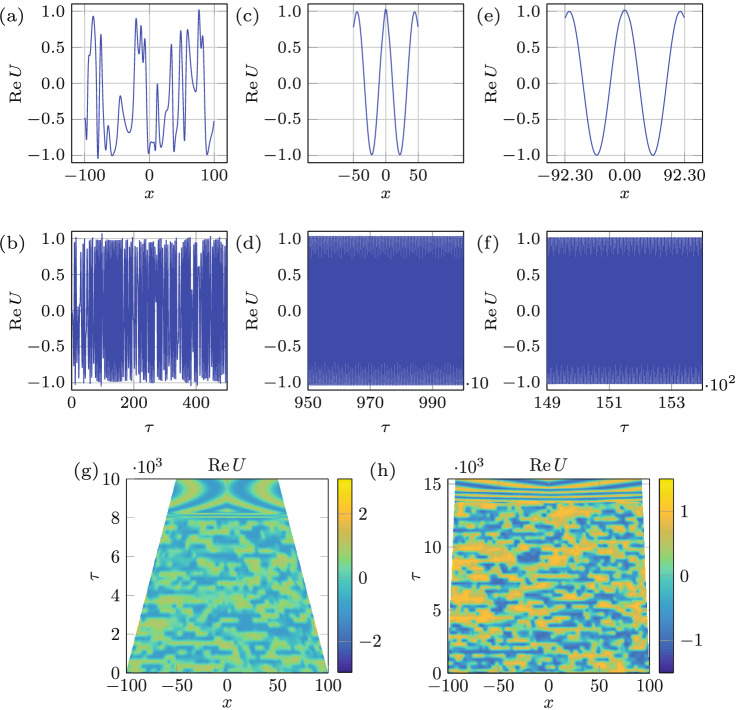
Domain evolution rate effect. (**a**, **b**) depict the spatio-temporal bichaos used as the initial state with domain size $L= 200$ and $(\alpha ,\beta )=(0.8,-1.4)$. The domain shrinkage $\Delta L=100.00$ and $\Delta L=15.40$, over which the regime changes from chaotic to periodic, with two different rates $v=10^{-2}$ and $10^{-3}$ corresponding to the middle (**c**, **d**) and right columns (**e**, **f**), respectively. (**g**, **h**) Space-time plots of the of the two processes indicating the transition over $\delta L=22.91$ and $\delta L=2.29$, respectively.

In the course of the above numerical investigation, we also observed that domain shrinkage is more effective at controlling chaos; for instance, the domain growth fails to regularize the stronger chaotic state presented in Fig. 8a,b compared to the weaker chaotic state in Fig. 6a,b, whereas domain shrinkage is able to make the system periodic starting from both the weaker and the stronger chaotic states. This behavior is again rationalized by the fact that early and hence more frequent phase-slips during domain shrinkage can control the stronger chaotic state better compared to delayed phase slips during domain growth, which tend to keep the system in its existing state.

### The effect of the domain evolution rate

**Figure 9:**
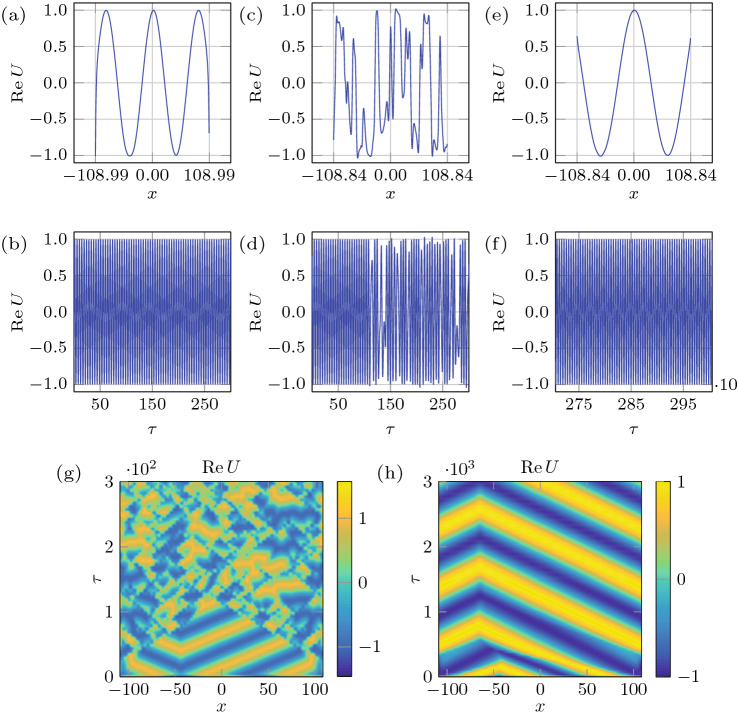
Domain evolution rate effect. The slower domain evolution prevents the system from entering the chaotic regime. (**a**, **b**) depict the initial periodic state (Fig. 6c,d). The domain shrinkage $\Delta L=0.3$ at two rates $v=10^{-3}$ and $10^{-4}$ correspond to the middle (**c**, **d**) and right columns (**e**, **f**) respectively. (**g**, **h**) Space-time plots of the two processes indicating transition over $\delta L=0.11$ and no transition, respectively.

Finally, a numerical study was performed to demonstrate the effect of domain evolution rate similar to experiments in Fig. 4: a periodic state that is reached by the domain growth (Fig. 6c) is used as the initial state for the domain shrinkage at two rates $v=0.001$ and $v=0.0001$ with the result reported in Fig. 9. While the faster rate (Fig. 9c,d,g) allows the system to go back to the expected chaotic state after shrinkage of $\delta L=0.11$ (similar to Fig. 6e,f), the slower rate (Fig. 9e,f,h) keeps the state of the system periodic even beyond the total domain shrinkage of $\Delta L=0.3$.

We also started from a chaotic state, rather than a periodic one, in order to see if the shrinkage rate would play a role in controlling chaos. Using as an initial condition a stronger chaotic state (Fig. 8a,b)—reached by picking the values $(\alpha ,\beta )=(0.8,-1.4)$ that are further away from the Benjamin–Feir–Newell curve—the total domain shrinkage required to enter the periodic regime at the rate $v=0.01$ is about $\Delta L \approx 100$ (Fig. 8c,d,g), whereas at the slower rate of $v=0.001$ the required total domain size variation is considerably smaller, $\Delta L=15.4$ (Fig. 8e,f,h). However, the transition itself again takes place only over a small portion of the domain evolution, i.e. $\delta L=22.91$ and 2.29 at the faster and slower rate, respectively (Fig. 8g,h). As alluded to earlier, at a lower speed the system is subject to smaller perturbations, the slower associated time scales of which allow the system to adapt and get attracted to a periodic orbit; at a higher speed the perturbations are larger thus making it harder for a system to get attracted to a stable periodic orbit, which statistically takes longer time and thus larger domain size variation. In other words, a slower rate facilitates the regularization of the regime, while a faster rate impedes the process: this is analogous to the experimental results on regular Faraday waves^29^ showing that pattern formation is naturally impeded during fast domain evolution as phase slips have no time to develop. Furthermore, these observations are consonant with the 1D theoretical findings^35^ indicating that more complex pattern sequences can be expected during slow domain evolution, which allows for more phase-slips to occur and therefore leads to changes sooner in the state of the system.

## Conclusions

In the presented work we demonstrated, both experimentally and theoretically, the ability to control chaos by the system size variation. These findings may shed some light on spatially evolving biological systems and life, that require ‘a healthy dose of chaos’ for proper operation^36^ and hence often balance on the edge of chaos as known from studies on cardiac and neuronal activity^37^, for example. The latter concept has also been encountered in many other areas^38^: in economy, creative destruction represents the driving force within a market economy; in social science, the dynamic interaction between individuals and macro-levels such as laws, religions, and governments imposing too much order and limiting individual development in the name of conformity, ultimately leading to stasis; in human cognition and creativity^39^, the states at the edge of chaos can be seen to be maximally novel while still connected to ones in the ordered regime—the hallmark of innovative thinking.

## Methods

### Apparatus

**Figure 10:**
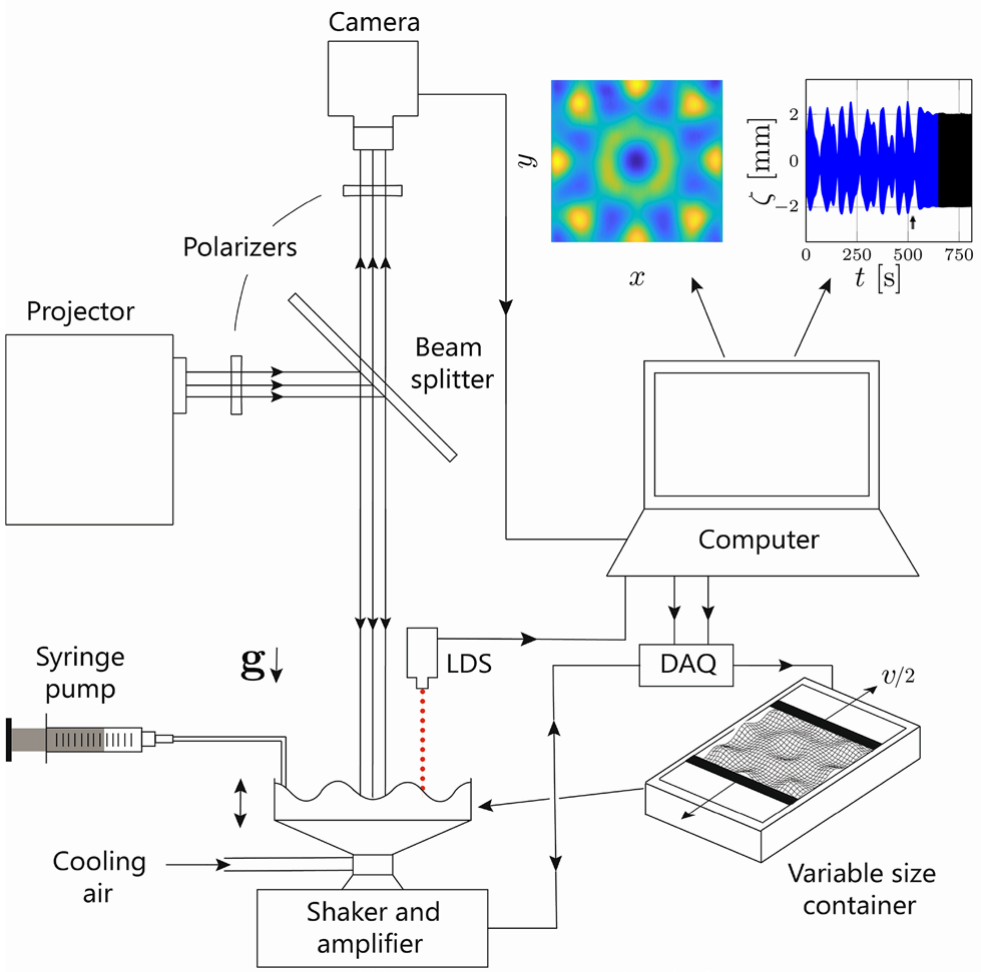
Experimental setup. The Faraday waves assembly with variable size container is mounted on the shaker which is driven using the amplifier and controlled through the data acquisition card (DAQ). The surface wave amplitude is measured using the laser displacement sensor (LDS). The camera, projector, beam splitter, and polarizers form the optical part required to visualize the Faraday waves.

The designed experimental setup shown in Fig. 10 produces Faraday waves and allows computer-controlled variation of the container dimensions in a time-dependent fashion as well as measurements of the formed pattern characteristics. The Faraday assembly is mounted on top of the electrodynamic shaker (Labworks ET-139) controlled by a computer signal via an amplifier. The liquid (water) is housed in a container with a transparent bottom and four sidewalls, so that inner tank dimensions are $150 \times 150 \times 12.7 \, {\mathrm {mm}}^3$ without moving walls. The length $L(\tau _{w})$ of the domain between the moving walls can be controlled in a time-dependent fashion according to the prescribed laws, where $\tau _{w}$ is a time scale longer than that of the vertical oscillations in order to avoid generating significant sloshing waves. Also, the liquid layer depth is kept constant first by allowing a clearance of about $2 \, {\mathrm {mm}}$ between the moving sidewalls and the bottom of the container, enabling unobstructed flow underneath the walls while not affecting Faraday waves^29^. Second, considering the sensitivity of Faraday waves to possible evaporation and thus to water layer depth changes^29,40^, in our setup the liquid level was also maintained constant at $h=12 \pm 0.1 \, {\mathrm {mm}}$ with the help of a syringe pump (PHD ULTRA Harvard apparatus) which injects water outside the moving walls at the rate $42 \, {\mathrm {\mu l \, min^{-1}}}$ required to compensate the evaporation for the existing conditions in the lab.

### Visualization technique

Figure 10 also schematizes the optical setup used to visualize Faraday waves with the help of the Fourier transform profilometry (FTP) technique—a single-shot optical profilometric measurement of surface deformation—which has been widely used in water wave studies^29,41,42^. This method is based on an optical system composed of a video projector (ViewSonic PJD7820HD) casting a grating pattern on the water free surface and a camera (Nikon D5200) recording the reflection of this pattern from the free surface. The grating pattern distorted due to deformation of the surface is then recorded and compared to the reference image of the undistorted grating pattern on the flat free surface in order to produce a phase-shift map—the difference between phases of light intensity at each pixel in these two images—from which the height of the deformed surface is reconstructed using a relation between the phase shift and the object’s height. The highly accurate common-optical axis implementation of FTP for water surface waves introduced recently^29^ guarantees a vertical resolution of $0.05 \, {\mathrm {mm}}$. For further details on the experimental setup and the visualization technique the reader may consult Ref.^29^.

**Figure 11:**
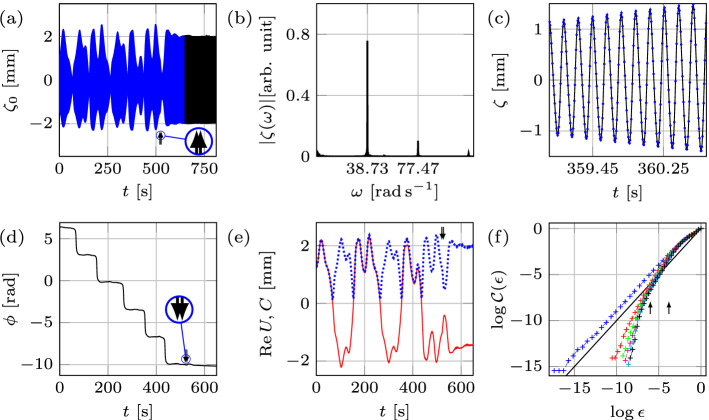
Data analysis procedure. (**a**) The recorded surface deformation $\zeta _{0}$ at the vessel’s centre with arrows indicating the start and finish of wall motion. The experimental conditions: $f=12.33 \, {\mathrm {Hz}}$, $A=1.16 \, {\mathrm {m \, s^{-2}}}$. The domain growth of $\Delta L= 2\, {\mathrm {mm}}$ starts from $L = 120 \, {\mathrm {mm}}$ at $t=520 \, {\mathrm {s}}$ with wall speed $0.15 \, {\mathrm {mm \, s^{-1}}}$. (**b**) Fourier transform’s amplitude $|\zeta _{0}(\omega )|$ of the recorded surface deformation. (**c**) Data points recorded by the laser for several fast cycles are presented along with the curves fitted to the data. (**d**) The extracted slow phase $\phi$. In (**e**) the amplitude envelope *C* and ${\text {Re}}U$ are shown by blue dotted and red solid curves, respectively. (**f**) The correlation function $\mathcal{C}(\epsilon )$ obtained from a single time series of ${\text {Re}}U$ for various embedding dimensions *m*. The solid black line with slope one signifies the saturated slope $\mathscr {D}$ in the scaling region (between the two arrows) is larger than one.

### Data analysis and chaos identification

For the present study, the setup reported in Ref.^29^ was modified in order to investigate temporal chaos. To that end, measurement of the waves amplitude at a single point on the surface is sufficient^7^ as explained below; hence, we resorted to a laser displacement sensor (Optex FA CDX-30A) capable of measuring the surface deformation with the accuracy of $0.01 \, {\mathrm {mm}}$ and the sampling rate of up to 80, 000 per second. Although no spatial information is provided by the measurement at a single point (2), combination with the Fourier transform profilometry used to visualize the free surface assured that there is no additional spatially-induced time dependence involved such as pattern rotation^7^.

An example of controlling chaos by domain deformation is provided in Fig. 11, which also illustrates the data analysis procedure. The surface deformation $\zeta _{0}(t)$ is recorded with the laser displacement sensor at the vessel’s centre (Fig. 11a), the Fourier transform of which (Fig. 11b) reveals that the strongest contribution is made by the Faraday waves oscillating at half of the driving frequency and the next contribution, though much weaker, comes from the meniscus waves.

Collecting data at the rate 150 per second and with a vertical resolution of $0.01 \, {\mathrm {mm}}$ enabled us to fully eliminate the meniscus wave contribution from the surface deformation. Following Eq. (1), the spatial contribution of the two modes $l_{1}$ and $l_{2}$ to the surface deformation at the centre of the container reduces to unity, i.e. $S_{l_{1}}=S_{l_{2}}=1$. Thus, with the inclusion of meniscus waves we arrive at equation (2). Given the Faraday waves total amplitude $a(t,\tau ) = C(\tau )\cos {\left[ \omega _{0} \, t+\phi (\tau )\right] }$, the complex slow amplitude is defined as

4 $$\begin{aligned} U(\tau ) = C(\tau ) \, {\mathrm {e}}^{- i \phi (\tau )}. \end{aligned}$$

The real and imaginary parts of $U(\tau )$ can be reconstructed from the measurements as follows. Using the recorded surface deformation given by (2), the amplitude envelope $C(\tau )$ (Fig. 11e), wave frequency $\omega _{0}$, and phase $\phi (\tau )$ (Fig. 11d) are recovered for each of the fast cycles individually sampled at the rate 25 data points per cycle, cf. Fig. 11c. Then, ${\text {Re}}U$ (Fig. 11e) and ${\text {Im}}U$ are determined based on equation (4).

Blocks of data were recorded continuously over a $650 \, {\mathrm {s}}$ interval, which is more than 20 times longer than required for the regime to change due to wall motion from periodic to chaotic or vice versa, also allowing us to carefully study the regimes before and after the domain deformation. Moreover, being capable to record continuously assured that the regime under investigation is not transient. For example, in Fig. 11d,e the extracted data are from the first block of $650 \, {\mathrm {s}}$ (shown with blue in Fig. 11a), covering $520 \, {\mathrm {s}}$ of the chaotic regime, the domain growth of $6.67 \, {\mathrm {s}}$ and the final periodic stage of $123.33 \, {\mathrm {s}}$. The second block of data—partially shown in Fig. 11a with black—is provided to confirm that the regime remains periodic. Finally, it should be noted that for different experiments, laser measurement naturally starts at an arbitrary instant within the single fast cycle period, resulting in a different initial phase $\phi$. To have the same initial phase $\phi$ for all the experiments, during the data analysis we picked up the starting data point of the first fast cycle to be at the same location during the fast cycle period for every experiment. Then, the reconstruction of the slow amplitude was initiated from the first fast cycle with a specific value of $\phi$, which in our case was selected to be $2.03\pi$ for the convenience of plotting.

To determine the dynamic type of the Faraday wave regime we resorted to the analysis of the total amplitude $a(t,\tau )$ using the embedding technique^43–45^, which with the measurement at a single location on the surface not only reveals the type of regularity of the regime (the dimension of the strange attractor) but also the number of modes involved. This technique assumes that all the important dynamical features are contained (embedded) in a single time series. A strange attractor is characterized as an aperiodic one, in which the surrounding trajectories diverge exponentially from each other in time, and, most importantly, is an object of fractal dimension *D*, i.e. the number of small cells of size $\epsilon$ required to cover the attractor scales as $\epsilon ^{-D}$ for $\epsilon \rightarrow 0$. The *m*-dimensional embedding phase-space coordinates are constructed as $\{{\text {Re}}U[t],\,{\text {Re}}U[t+\delta t],..., {\text {Re}}U[t+(m-1)\delta t]\}$, where $\delta t$ is an arbitrary time delay. The theory^43,45^ assures that the topological properties extracted from the above embedding, such as dimension and Lyapunov exponent, are equivalent to that of the attractor provided that $m \ge 2D+1$. Practically, it is very difficult to measure *D* from an experimental data set^6,7,46,47^; however, there exist equivalent estimates of *D*, the most common of which is based on the correlation dimension:

5 $$\begin{aligned} {\mathscr {D}} = \lim _{\epsilon \rightarrow 0}\frac{\log {\mathcal{C}}(\epsilon )}{\log \epsilon } \le D, \end{aligned}$$

where the correlation function ${\mathcal{C}}(\epsilon )$ is the number of data-point pairs separated by a distance shorter than $\epsilon$ in the phase-space multiplied by $N^{-2}$ with *N* denoting the total number of data points. The numerical implementation of (5) has been well developed in literature^6, 47,48^. Once ${\mathcal{C}}(\epsilon )$ is determined and plotted against $\epsilon$ for different values of the phase-space dimension *m*, the limiting slope in the scaling region (e.g. in Fig. 11f it is the neighborhood of $\log (\epsilon )=-5$ shown by the arrows) defines the value of ${\mathscr {D}}$. It can be seen from Fig. 11f that the slope in the scaling region does not change with further increase of *m* beyond four and is saturated at the value of $1.82\pm 0.02$, which corresponds to the fractal dimension ${\mathscr {D}}$ of the strange attractor thus indicating that the system is in a chaotic state and can be described by a 4-dimensional phase-space formed by real and imaginary parts of the two slow amplitudes.

## Data Availability

All data needed to evaluate the conclusions in the paper are present in the paper and/or the Supplementary Materials. Extended data, software, and materials are available upon request by contacting the corresponding author.
